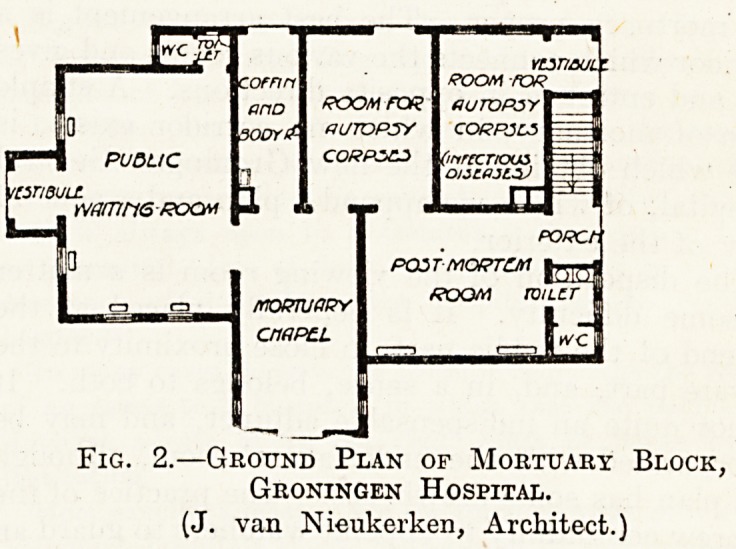# The Mortuary Unit

**Published:** 1913-03-01

**Authors:** 


### THE MAKING OF A MODERN HOSPITAL.

### The Mortuary Unit.

### THE PUBLIC PART OF THE MORTUARY.—III.

By the public part of a mortuary we mean that portion of the block which is accessible to the friends and relatives of the patients, to the undertakers, and those who are responsible for the taking away and burial of the corpse, and generally for those who have, strictly speaking, no interest in visiting the post-mortem room and the laboratories attached

Fig. 1.—Exterior View of Mortuary Block. Groningen Hospital.

to it. These latter, for convenience of description, we shall term the private part of the mortuary block.

The dimensions and construction of the public part will depend largely on the size of the hospital or institution and on the work to be done in the part. Abroad, where medico-legal work is carried on at many hospitals, the latitude allowed to the architect in drafting the plans for the public portion of the mortuary is considerably greater than with us, where most of the legal post mortems are carried out in public mortuaries. At the London Hospital, however, there is provision made for the Coroner's Court, and at a few provincial institutions similar provision exists. This, in our opinion, is a mistake. The public mortuary should be housed in a separate building, and should be wholly in charge of public officials. At present it is the custom for the police to take a corpse to the nearest mortuary, and a dying man found on the streets to the nearest hospital. We believe that hospital residents rather encourage the latter practice. In fact, to the average unpaid house surgeon or house physician at one of our large hospitals, a B.I.D. (brought in dead) is in the nature of a godsend, the reason being that when a resident is called upon to give evidence in a Coroner's Court respecting the case of a patient who has died outside the limits of the charity to which the witness is attached, the legal fee of one guinea is paid to him. Residents cannot claim such a fee in the case of a patient who has died in hospital. To the resident, therefore, it is a matter of some concern whether the patient has breathed his last on the ambulance outside the hospital gates or whether he has expired in the receiving room; in the former case he is a B.I.D.; in the latter, technically at least, a hospital—that is, charity—patient. We are not now concerned with the unfairness of this distinction, which has often been commented upon by Coroners, but we merely wish to point out that so long as the present system lasts hospital residents will most likely continue to encourage the practice of receiving B.I.D.s, and turning their mortuaries into public mortuaries for the sake of the witness and autopsy fees that they may claim. In the interests of the hospitals this practice is reprehensible and should be discouraged, for the obvious reason that the hospital mortuary is an examining station, which is as much a part of the scientific side of the institution as the dispensary or the laboratory.

Considering, then, the hospital mortuary as a place solely intended for the patients who die in the institution itself, as an institutional and not as a public department, we conclude, from what has been said in preliminary articles regarding the desirability of safeguarding the public interests, that adequate provision must be made for the patients' friends and relatives. The least possible provision includes a viewing room, a waiting room, a private room where such preliminary obsequies as the friends may desire can conveniently be carried out, and a lava-

Fig. 2.—Ground Plan of Mortuary Block, Groningen Hospital. (J. van Nieukerken, Architect.)

tory. To this, strictly speaking, we must add an undertakers' room, but at a pinch this may be relegated to the private part, though in our opinion it is much better to draw a sharp line of demarcation between the public and private portions of the block and to admit to the latter none who have not an institutional interest. The undertaker's work is obviously not institutional, and it is therefore best to supply him and his assistants with a small room, opening out of the viewing room, wherein they may coffin the corpse in strict privacy. This room need not be large, though it should be commodious enough not to interfere with the needful manipulations. All the furniture it should contain are a couple of trestles, a small table for tools and implements (these are generally supplied by the undertaker, but a set should be kept in the block for use when required), and a cupboard with hooks for coats and hats. Proper ventilation and warmth must be secured in this room, and for ease of cleansing a tiled wall and floor are desirable. As a matter of fact it may be taken as a rule that in every part of the mortuary where the uncoffined dead body may rest, the floor at least should be of some impervious, easily cleansible material. A good cement floor usually serves well; although it is not, strictly speaking, impervious, yet it absorbs odours and fluids far less easily than composition floors or lino. Mosaic floors and tiles are less satisfactory, for one reason because the interstices, however evenly the floor may be laid, are apt to absorb droppings; and, secondly, because the composite fragments sometimes work loose, especially if the floor is vigorously scrubbed. A good but comparatively expensive floor is one made of large marble slabs, laid in cement with the joints closed by some impervious composition. But for all practical purposes a good cement floor, properly looked after, and with adequate slope and arrangements for flushing, is on the whole the most satisfactory. The slope should never be more than just sufficient to carry down the room the flow of a half-inch pipe at ordinary water pressure left running for a few seconds; a greater slope is quite unnecessary, and although it does not in the mortuary give rise to the inconveniences which its presence entails in the operating theatre, it serves no useful purpose, and should be avoided. The entrance to the public part, as we have already stated, must be separate and distinct from that of the mortuary proper. The best arrangement is a corridor which connects the various rooms and gives exit and entrance in opposite directions. A simple form of mortuary, in which no corridor exists, is that which obtains at the new Groningen General Hospital, of which we append a plan and a general view of the exterior.

The disposition of the viewing room is a matter of some difficulty. It is generally placed at the far end of the public part, in close proximity to the private part, and, in a sense, belongs to both. It is not quite an indispensable adjunct, and may be incorporated with the undertakers' room, although this plan has some drawbacks. The practice of the Hebrew community to appoint watchers to guard an unburied corpse has to be taken into consideration in hospitals which admit Jewish patients. Facilities for such guards to watch the dead may be provided either in the viewing room or in the mortuary itself by a special "squint" which admits of a glimpse into the chilling room or into the institutional part. With this point we shall deal more fully in our next article. The friends' waiting room should be arranged at the other extremity of the corridor, away from the undertakers' room, and isolated from the private part, so that those who are in it may be quite undisturbed by the sounds or sights around them. The prevailing tendency is to make this room bare and comfortless, a tendency which is neither in the interests of the institution concerned nor in those of the relatives and friends. The room should be plainly but comfortably furnished, well lighted and warmed, and ventilated. Here a lino floor on a cement foundation is preferable to any other kind of floor, except perhaps a hard wood one; it gives a more cheerful appearance than a mosaic floor, is less noisy, and is warmer, while on the score of cleanliness there is little to object to. The walls should be of plain distemper, warm but not sombre in colour, and if a few suitable pictures, carefully chosen, are arranged on them so much the better. A plain uncovered table, a comfortable wooden settee, and half a dozen comfortable wooden chairs, together with a large-face timepiece, are all the furniture necessary. The provision of hat or coat racks in the waiting room is unnecessary, but an umbrella stand, with zinc or metal bottom, for the reception of wet umbrellas, should be provided. We have already dealt with the provision for lavatory accommodation in connection with the waiting room; all that need be added is that access to such lavatories should be easy from the waiting room, and that proper indications should be given where they are.

The private room is generally dignified with the name of the hospital mortuary chapel, and in some institutions is a most elaborate chamber, built at a cost far exceeding its utility. This is notably the case at the Virchow Hospital, where the mortuary chapel, which stands somewhat apart, is one of the features of the institution. The Munich-Schwabing Hospital has even gone to a greater expense and has provided two magnificent chapels, one for the Catholic community and the other for the Protestant. Where religious differences are so great that the hospital authorities have to take them into consideration, and where private charity is willing to come to the support of the institution to build and furnish such chapels, little can be said against them; but in ordinary cases the institution cannot be called upon to erect separate chapels for the various religious denominations. The funeral obsequies carried out in the block, in ordinary circumstances, must be in the nature of preliminaries, and the chapel must be regarded as common ground. But that consideration does not alter the fact that it is in the interests of the hospital to provide a room, which, whether it be large enough and elaborate enough to be styled a chapel or not, is at least attractive and dignified. A little expense spent on furnishing and beautifying this apartment will be amply repaid by the prestige that the institution secures from the possession of a chapel which symbolises that reverence and respect for the dead which the public have a right to expect. The type of building and the nature of its adornment must be left to individual taste and judgment. In Italian hospitals, which are not conspicuous by their modernity or perfection of construction, the visitor is usually struck with the minute attention which has been paid to the mortuary chapel—an attention which does not always mean the expense of so much money, but generally the kindly care of friends and benefactors inspired by a devotional love and reverence. By raising the roof, by introducing some design in walls and windows, by the addition of coloured glass, and by other means much may be done to make the private room essentially different in character and type from the working rooms of the block.

## Figures and Tables

**Fig. 1. f1:**
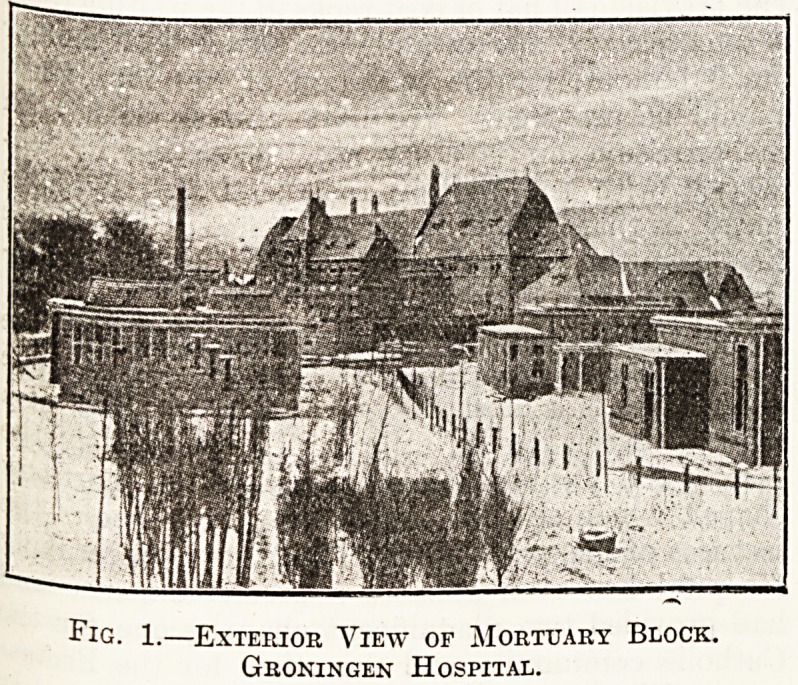


**Fig. 2. f2:**